# From Individual to Group: Organizational and Relational Strategies for Nurse Leader Well‐Being

**DOI:** 10.1155/jonm/7568215

**Published:** 2026-08-23

**Authors:** Jan Heiberg Johansen, Malene Friis Andersen

**Affiliations:** ^1^ Department of Society and Politics, Aalborg University, Aalborg, Denmark, aau.dk; ^2^ Psychosocial Working Environment, National Research Centre for the Working Environment, Copenhagen, Denmark, arbejdsmiljoforskning.dk

## Abstract

**Aim:**

This study identifies psychosocial risk and resource factors influencing nurse leaders’ well‐being at the relational and group levels to guide organizational strategies for improvement.

**Background:**

Nurse leaders face significant emotional and organizational demands that strain their well‐being. Research on interventions to support nurse leaders’ well‐being has largely focused on individual strategies, with less attention to relational and organizational factors. This study addresses that gap by examining how leadership groups and relational dynamics influence nurse leaders’ well‐being. Understanding these influences is crucial for improving nurse leaders’ well‐being and reducing stress.

**Methods:**

The study employs a qualitative, exploratory design with 22 semistructured interviews with 18 nurse leaders and four other health managers working together in interprofessional leadership groups from a Danish healthcare region. The interview guide and analysis were informed by prior research on nurse leader well‐being and by the job demands–resources and conservation of resources frameworks. Using thematic analysis, we identified key patterns of relational risks, resources, and strategies, guided by these frameworks.

**Results:**

Participants described risks and resources across four relational and organizational levels: the immediate leader, the peer leadership group, the broader leadership network, and the work system surrounding the leadership group and leader. We identified key risk and resource factors on each level. Risks included weak supervisory support, low peer trust, isolation, and being alone with heavy workloads. Resources encompassed supportive supervisors, peer safety within leadership groups, access to mentoring and reflection across units, and manageable workloads. Suggested relational and organizational strategies focused on fostering peer safety, implementing coleadership or buddy systems, protecting leadership time, ensuring influence in decision‐making, and creating cross‐unit forums for support and peer supervision.

**Implications for Nursing Management:**

The findings identify four relational and organizational levels, and the implications address them. Organizations should establish (1) clear priorities, transparent involvement in decisions, and visible backing from immediate leaders; (2) leadership groups as peer‐reflection forums for systematically addressing leadership dilemmas; (3) cross‐unit mentoring, buddy arrangements, or other confidential professional support; and (4) a regular review of structural factors such as spans of control, administrative workload, protected leadership time, and expectations of after‐hours availability.

**Conclusion:**

The findings identify supportive leader groups, trusting supervisory relationships, and well‐structured work systems as resources for nurse leaders’ well‐being. This study introduces nurse leader well‐being as an organizational capability that emerges from relational structures and organizational strategies.

## 1. Introduction

Nurse leaders’ well‐being has effects that extend beyond the individual leader, shaping patient outcomes and the work environment for frontline staff [1, 2].

The well‐being of nurse leaders affects quality of care and organizational performance [3], while lack of adequate support for nurse leaders’ well‐being has been linked to adverse downstream effects on staff and patient outcomes [1, 4, 5].

Recent research shows that leaders under strain may withdraw from proactive leadership, delay decisions, or struggle to remain emotionally composed, which can weaken coordination, communication, and long‐term improvement efforts [6–8]. Exhausted leaders may lack the capacity to focus on quality improvement and team development, whereas supported leaders are better equipped to contribute positively to organizational performance [2, 9]. Therefore, addressing the well‐being of nurse leaders may be an overlooked factor in enhancing overall healthcare delivery.

Empirical knowledge on how to enhance leader well‐being often remains individual‐focused [10]. Prior research has explored stress, burnout, and coping among nurse leaders as personal issues [11, 12], with interventions centered on individual‐level stress management or mindfulness [13]. However, recent studies show that nurse leaders’ well‐being is deeply influenced by relational and organizational factors, including workload demands, insufficient organizational support, and the quality of peer and supervisory relationships [1, 2, 14, 15]. These contextual elements not only affect leaders’ psychological health but also their ability to foster healthy, sustainable work environments for others [9, 16].

Nurse leader well‐being is shaped by the balance between demands and resources in the psychosocial work environment. The job demands–resources (JD–R) model distinguishes between job demands, such as workload, time pressure, role conflict, and emotional effort, and job resources, such as autonomy, influence, support, and opportunities for recovery [17]. This perspective aligns with conservation of resources (COR) theory, which emphasizes that stress arises when valued resources are threatened, depleted, or insufficiently replenished over time [6, 18, 19].

Research on nurse leaders identifies high workloads, large spans of control, role conflict, competing priorities, and emotional demands as key contributors to stress, burnout, and turnover intentions [4, 9, 11, 20, 21]. At the same time, autonomy, influence, organizational support, supervisory support, peer support, and access to professional networks have been linked to job satisfaction, retention, and improved work‐related well‐being among nurse leaders [2, 14, 22, 23].

Although this literature identifies important risk and resource factors, interventions for nurse leaders’ well‐being have often emphasized individual‐level stress management rather than relational or organizational conditions [13]. Less is known about how leadership groups function as everyday settings in which demands are shared, interpreted, and managed and in which resources such as trust, peer support, and collective reflection may be present or absent. This creates a need for empirical research that examines nurse leaders’ well‐being as a relational and group‐level phenomenon.

To address this gap, we conducted a qualitative interview study examining how nurse leaders describe risk and resource factors in their relational and organizational work environments. In this study, the term nurse leader refers to first‐ and middle‐level nurse leaders with formal responsibility for nursing staff and units.

The study was guided by the following research question: What are the key risk and resource factors affecting nurse leaders’ well‐being at the relational and group levels, and how can we use this knowledge to improve organizational strategies to enhance their well‐being?

The article is structured as follows: First, we outline the methodological approach; next, we present the empirical findings; and finally, we discuss implications for theory and practice.

## 2. Methods

We conducted an exploratory qualitative study to examine how nurse leaders describe risks and resources in their psychosocial work environment. A qualitative design was appropriate because the study explored complex, context‐dependent experiences for which empirical knowledge of relational and group‐level processes remains limited [24]. Individual semistructured interviews were selected because they combine a shared interview structure with the flexibility to follow up on issues participants considered particularly relevant to their leadership work and well‐being [25].

The interview guide was informed by prior research on nurse leader workload, role stress, autonomy, supervisory support, peer support, and coping [2, 11, 14, 22] and by the JD–R distinction between demands and resources [17]. This prior literature served as a sensitizing background for developing interview topics and for interpreting risk and resource factors. The study is reported in accordance with the Consolidated Criteria for Reporting Qualitative Research (COREQ) guidelines for interview‐based studies [26]. Please refer to the study’s Supporting Information for a full COREQ overview.

### 2.1. Setting and Participants

The study was conducted in a Danish healthcare region with more than 1200 leaders primarily working in hospital and social service settings. We used purposive sampling to include leaders with direct experience in everyday healthcare leadership and to capture variation in leadership level, department type, professional background, and tenure. Participants were eligible if they (1) held a formal leadership role with personnel responsibility, (2) were currently employed in the regional healthcare system, and (3) had direct experience leading staff or other managers.

The sample consisted of 22 leaders, including 18 nurse leaders and four other health managers who worked in interprofessional leadership groups with nurse leaders. Participants included first‐line leaders, responsible for frontline staff and nursing units, and middle‐level leaders, who managed other leaders or larger departments. Top‐level executives were excluded because the study focused on relational and group‐level conditions in daily leadership work among first‐line and middle‐level leaders. The participants worked alongside other healthcare leaders from different professional backgrounds, often within leadership groups that strongly influence the psychosocial work environment of nurse leaders [8, 14].

Participants came from various departments, including medical, surgical, and psychiatric, with leadership tenures ranging from about 2 to 25 years, most with 5–10 years of experience. Fourteen participants were women, and eight were men, reflecting some gender diversity among the leaders. To recruit participants, we partnered with the regional nursing leadership network to invite volunteer participants. Participation was kept confidential.

Sample size was guided by the principle of information power, which considers the specificity of the study aim, sample characteristics, use of theory, quality of dialog, and analytic strategy [27]. Information power was strengthened by the focused research question, the inclusion of participants with direct leadership responsibility within the same regional healthcare system, the use of JD–R/COR as sensitizing frameworks, and the depth of the semistructured interviews. During data collection, the authors discussed interview summaries, emerging codes, and whether new interviews added substantively new risk or resource factors. After approximately two‐thirds of the interviews, no new major risk/resource categories or relational levels were identified; the remaining interviews mainly refined, nuanced, and confirmed the developing themes. This was treated as thematic saturation in line with Guest et al. [28].

### 2.2. Data Collection (Interviews)

**FIGURE 1:**
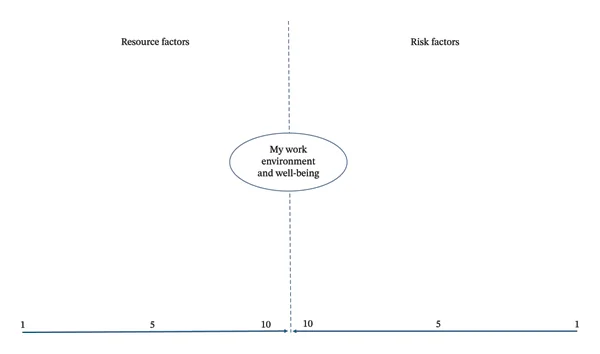
Interview model.

Data were collected through one‐on‐one semistructured interviews conducted in March 2025. An interview guide focused on risk and resource factors in the nurse leader’s work environment. The guide was developed from prior research on nurse leader well‐being, ensuring alignment between existing evidence and the empirical inquiry. To provide structure and ensure comprehensive coverage, we introduced a straightforward analytical model during the interviews: a visual diagram with “My work environment and well‐being” at the center, flanked by two columns labeled Resources and Risks (Figure 1). Participants were asked to identify and discuss factors in each column as the interviews progressed.

Participants were asked broad, open‐ended questions, such as the following: “What aspects of your work make it hard for you to thrive or feel well as a leader?” (to identify risk factors) and “What factors help you maintain your well‐being in your leadership role?” (to identify resource factors). Additional probes examined relationships with staff and fellow leaders, coping strategies, and work–life balance. Participants were prompted to provide concrete examples of experiences with resource and risk factors to facilitate the collection of detailed data [29].

The interviewer recorded notes in real time using the interview model (Figure 1), listing factors identified by participants and asking them to rate each factor’s impact on their well‐being on a scale from 1 (*very low impact*) to 10 (*very high impact*). This interactive approach helped participants systematically reflect on risks and resources for their well‐being. The interviews lasted approximately 60 min, were audio‐recorded, and were transcribed verbatim. Additionally, respondents were asked to consider potential strategies and interventions aimed at nurse leaders’ well‐being.

### 2.3. Data Analysis

**FIGURE 2:**
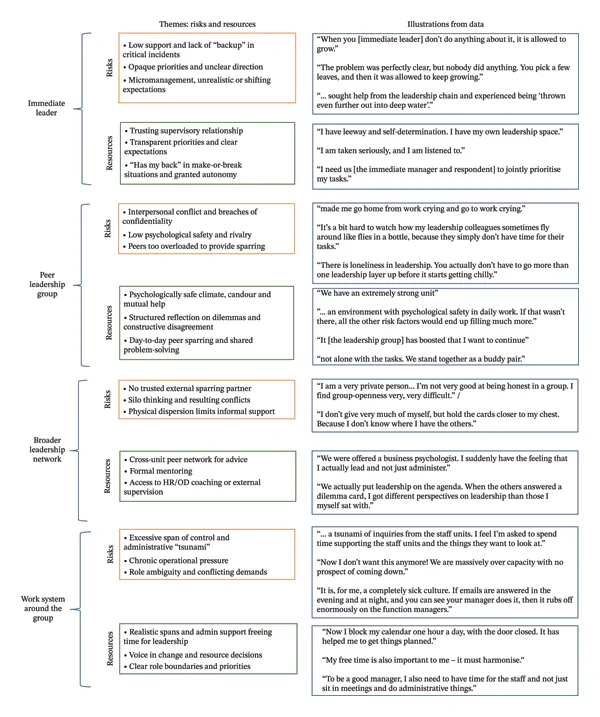
Overview of data analysis.

We used thematic analysis to identify patterns in the interview data [30]. The analysis was primarily data‐driven, with the JD–R distinction between job demands and job resources serving as a sensitizing framework for interpreting participants’ descriptions of risks and resources [17]. The data analysis consisted of three steps: (1) reading and rereading transcripts to familiarize ourselves with the data; (2) initial coding of segments relevant to nurse leader well‐being, highlighting text that indicated specific risks or resources, with particular attention to participants’ highest‐ranked themes; and (3) grouping codes into candidate themes under the broad categories of “Risk Factors” and “Resource Factors” (Figure 2). We used both the theoretical framework as an organizing guide and the interview model to focus on themes that received consistently high ratings across respondents.

Two researchers (the authors) independently coded five interview transcripts to enhance analytic consistency and reflexivity. They then compared and refined the code definitions, reaching consensus on the main themes. The entire dataset was subsequently coded using the agreed‐upon coding scheme. We summarized each participant’s reported risk and resource factors using the interview model (Figure 1), which helped identify the most frequently and strongly endorsed factors among the 22 leaders. Regular meetings between the authors were held to discuss emerging themes and resolve any discrepancies in interpretation, improving the trustworthiness and consistency of the analysis. Representative quotes from the interviews were selected to illustrate each theme. Quotes were anonymized by removing identifying details, such as specific job titles or departments, to protect confidentiality.

The authors have backgrounds in organizational psychology and leadership research, as well as prior experience in healthcare settings. Recognizing that this positioning could bias interpretations toward relational explanations, the authors addressed this by employing joint coding, systematically considering alternative interpretations, and engaging in ongoing analytic discussions in which assumptions and interpretive frameworks were explicitly identified and critically examined [31].

### 2.4. Ethical Considerations

The study adhered to relevant national and institutional ethical guidelines. Formal review by the Danish scientific ethics committee system was not required under Danish regulations because the study examined organizational aspects of working life and did not constitute a health science research project.

Institutional permission to conduct the study and recruit participants was obtained before data collection began. Participants provided informed consent before participating. We ensured confidentiality and anonymized reporting to encourage honest reporting of both positive and negative experiences. No individual or hospital unit is identified in the results. We do not specify participants’ exact roles or locations, and quotes are anonymized. At the end of the study, a summary of findings was shared with the regional nursing leadership council to disseminate knowledge and support the community.

## 3. Findings

The findings are based on interviews with 22 healthcare leaders, including 18 nurse leaders and four other health managers who worked closely with nurse leaders in interprofessional leadership groups. Participants held first‐ or middle‐level leadership positions with personnel responsibility. They worked across hospital and social service settings, including medical, surgical, psychiatric, and other departments. Leadership tenure ranged from approximately 2 to 25 years, with most participants having 5–10 years of leadership experience. Fourteen participants were women, and eight were men. To protect anonymity, we do not report more detailed combinations of role, department, gender, or tenure.

The findings are organized around four relational and organizational levels that participants identified as important to their well‐being. Participants described both risks and resources at all four levels. The four levels are as follows: (1) the immediate leader, (2) the peer leadership group, (3) the broader leadership network (including cross‐unit peers, mentors, and HR/OD), and (4) the work system surrounding the leadership group and leader.

**FIGURE 3:**
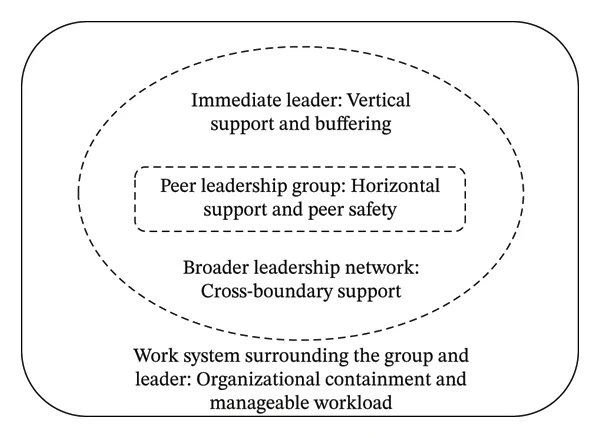
Four relational and organizational levels in nurse leaders’ psychosocial work environment.

Figure 3 presents the four levels used to structure the findings. Within each level, we present risk and resource factors reported by participants and illustrate them with anonymized quotations.

### 3.1. Immediate Leader

#### 3.1.1. Risks in the Relationship With the Immediate Leader

Throughout the interviews, the relationship with the immediate leader was described as a “critical source of stress” when support was lacking or absent, especially in difficult work situations perceived as critical or in escalating conflicts. Several leaders shared experiences in which work problems were clearly identifiable but not addressed by higher leadership, allowing them to worsen over time: “When you [immediate leader] don’t do anything about it, it is allowed to grow.”

One participant explained, “The problem was perfectly clear, but nobody did anything. You pick a few leaves, and then it was allowed to keep growing.” Leaders described how unresolved issues were permitted to fester rather than being addressed early. Such a lack of support or “opaque” guidance from one’s immediate leader left participants feeling “weak in the hierarchy,” unsure of priorities, and “vulnerable to overload.”

Leaders also described situations in which they actively sought support from their immediate leader during challenging times, only to face increased exposure and vulnerability. One participant summarized this experience as, “I was thrown even further out into deep water.” These experiences were linked to feelings of standing alone and being weakly positioned within the hierarchy. Participants described these situations as especially difficult when they involved issues they regarded as critical to their role.

In addition to lacking support during critical incidents, leaders cited unclear priorities and vague direction from their immediate leader as contributors to stress. Others identified shifting or overly high expectations, micromanagement, and perceived unfairness or favoritism, which eroded trust and were described as adding pressure in challenging situations.

#### 3.1.2. Resources in the Relationship With the Immediate Leader

Leaders described their immediate leader as an important resource when the relationship was marked by trust, care, and clear expectations, noting that the leader “has my back.” Several participants emphasized the importance of autonomy rooted in a “supportive supervisory relationship,” as one leader put it, “I have leeway and self‐determination – I have my own leadership space.” Others emphasized feeling recognized and included by upper management: “I am taken seriously, and I am listened to.” Another leader stated, “I feel that I am included and supported by my manager and director.”

Support from the immediate leader was especially emphasized in situations deemed “critical.” In these cases, “visible backing” and shared responsibility were described as stabilizing and important for leaders trying to cope and continue in their roles.

Feeling “invited inside” major decisions by immediate leaders or top management was described as increasing commitment and easing tension: “When I feel being utilized. I am taken seriously, and I am listened to. That is super great.” Being given the opportunity to participate in strategic decisions signaled recognition and gave participants a greater sense of control. Meanwhile, leaders cautioned that uneven or unclear distribution of opportunities could lead to “envy or disharmony in relationships with your managerial colleagues,” highlighting the importance of inclusive processes and transparent communication from immediate leaders to sustain trust within the leadership group.

Clear and transparent priorities, along with granted autonomy and decision‐making freedom, were repeatedly described as conditions that leaders associated with less uncertainty and more effective leadership under pressure.

### 3.2. Peer Leadership Group

#### 3.2.1. Risks Within the Peer Leadership Group

Throughout the interviews, the peer leadership group emerged as a key relational setting where leaders either found support or experienced additional stress. Several nurse leaders shared stories of conflicts within the leadership group that became personal, “breaking the collegial bond,” and where the peer group could turn into a source of “distress rather than support.” Participants highlighted experiences in which breaches of confidentiality made them reluctant to seek help. One leader reflected on the emotional toll of such situations: “I went home from work crying and went to work crying.”

One leader learned that confiding in a peer could backfire when something she shared in confidence later resurfaced to her disadvantage. She described feeling that “someone knows something I thought was confidential.” The experience made her cautious, and she said she would “keep my cards close to my chest because I do not know where I stand with the others.” Such breakdowns in peer confidentiality were described as weakening the sense of support.

Others emphasized that a lack of time and peer workloads were limiting opportunities for mutual support and sparring. One participant described seeing colleagues under pressure: “They fly around like flies in a bottle.” In these cases, leaders expressed feeling unable to rely on peers without adding to their burden, and participants described a sense of isolation. Participants reflected this by mentioning, for example, “a lonely posting,” “standing alone,” or stating that “there is loneliness in leadership,” as well as noting that relationships could cool down.

Across these accounts, silo dynamics and peers’ lack of capacity to support one another were described as factors associated with weaker horizontal relationships and with leaders feeling more isolated when facing tough decisions and emotional stress.

#### 3.2.2. Resources Within the Peer Leadership Group

In contrast, leaders also described the peer leadership group as a vital resource when relationships were marked by trust, candor, and mutual support. Participants collectively emphasized the importance of “daily interactions” and “helping each other,” which allowed concerns, doubts, and disagreements to be voiced openly. One leader described such a climate as foundational for daily leadership work: “We have an extremely strong unit. We have created an environment with psychological safety in daily work. If that was not there, all the other risk factors would end up filling much more.”

Others highlighted the value of honest dialog and constructive disagreement within the group. One participant emphasized that open disagreement was seen as supportive and developmental rather than threatening: “We can be ragingly disagreeing. I like that.” Daily peer sparring and shared problem‐solving were described as especially important for managing workload and dilemmas. Leaders noted that feeling supported by peers lessened the sense of being alone with tasks and responsibilities. Some participants had formal coleadership pairs (e.g., between a head nurse and a chief physician) that provided ongoing support and motivation. As one participant expressed it, “It has boosted that I want to continue [in my job]. You are not alone with the tasks; we stand together as a buddy pair.” Others described lighter‐weight “go‐to” buddies who could step in during peak pressure or fill in when needed.

Within the leadership group, formalized discussion of management dilemmas at meetings was also described as an important resource. Several leaders reported deliberately bringing leadership issues and dilemmas into team meetings to enable shared reflection. These practices were described as opportunities for collective sense‐making and mutual support amid the uncertainties of leadership work.

### 3.3. Broader Leadership Network

#### 3.3.1. Risks in the Broader Leadership Network

Across interviews, leaders described the broader leadership network beyond their immediate peer group as an important yet often fragile source of support. When access to cross‐unit peers, mentors, or external sparring partners was limited, leaders reported a narrowed perspective and a heightened sense of being “alone at the top.” Several participants described difficulty being open in broader group settings, which they said limited their ability to use these forums as sources of support. One leader explained, “I am a very private person. I am not very good at being honest in a group. I find group‐openness very, very difficult.”

Some leaders discussed an “old” and a “new” culture, with less‐experienced nurse leaders highlighting “silo thinking” among more experienced colleagues, in contrast to “we thinking” and collaboration across departments to succeed as a hospital. These experiences were associated with frustration about cross‐organizational collaboration with leaders outside their own peer leadership group. As a result, even when formal cross‐organizational networks existed, they might not provide spaces for reflection or emotional relief. Physical dispersion across locations was described as reducing opportunities for informal interactions and spontaneous exchanges and limiting access to alternative perspectives on leadership challenges.

These accounts describe circumstances in which the broader leadership network was perceived as not providing adequate support, and leaders reported having limited safe spaces for reflection outside their immediate organizational context.

#### 3.3.2. Resources in the Broader Leadership Network

Leaders described cross‐boundary networks as valuable resources that provided access to trusted sparring partners and viewpoints outside their own units. Several participants emphasized the importance of cross‐unit peer networks for advice and benchmarking and for comparing experiences and normalizing challenges. Formal mentoring relationships were especially valuable, particularly when more experienced leaders supported newer or at‐risk leaders. In some parts of the organization, this was implemented by assigning a more experienced leader from another department as a buddy to new leaders, who could offer sparring and provide feedback from an external perspective.

Access to HR, organizational development, or external coaching was also described as a valuable resource. Leaders emphasized the value of having a sparring partner with “nothing at stake,” which encouraged more candid reflection. One participant noted, “I have gotten a great deal out of using [a colleague] from HR, because he has nothing at stake.” Others described how external professional support helped them transition from feeling overwhelmed by administrative tasks to reconnecting with their core leadership role. One leader reflected, “We were offered a business psychologist. I suddenly have the feeling that I actually lead and not just administer.”

Structured cross‐boundary reflection forums were also described as supportive. Leaders valued settings where leadership dilemmas could be discussed openly and collectively, yielding new perspectives. One participant explained how these practices expanded their understanding: “We actually put leadership on the agenda. When the others answered a dilemma card, I got different perspectives on leadership than those I myself sat with.” These experiences were described as broadening leaders’ interpretive frames and lessening the sense of facing challenges alone.

### 3.4. Work System Surrounding the Group and the Leader

#### 3.4.1. Risks in the Work System Surrounding the Group and the Leader

Across interviews, leaders described the surrounding work system as a condition that could either add to stress or support within leadership groups. When structural conditions were perceived as excessive or poorly managed, even strong relational resources were deemed inadequate. Several participants identified large spans of control and increasing administrative demands as the primary sources of pressure and described a sense of “depletion” and “difficulty coping with the full management task.” One leader described the situation as overwhelming: “We are experiencing a tsunami of inquiries from the staff units at the hospitals… I feel I’m asked to spend time supporting the staff units and the things they want to look at.”

Others highlighted “high workloads” and noted that working chronically over capacity, as described by participants, was associated with a sense of futility and fatigue. One participant said, “Now I don’t want this anymore! We are massively over capacity with no prospect of coming down.” Such conditions felt as if they left “little room” for recovery, reflection, or leadership beyond firefighting. Role ambiguity and conflicting demands added to this pressure as leaders tried to balance expectations for administration, personnel management, and operational delivery.

Leaders also described organizational cultures that implicitly promoted overwork. One participant found this deeply problematic: “It is, for me, a completely sick culture… if emails are answered in the evening and at night, and you can see your immediate leader does it, then it rubs off.” In these accounts, norms of constant availability and responsiveness were described as spreading through the hierarchy, increasing stress and making it harder for leaders to set boundaries. Overall, these structural and cultural factors were described as increasing demands and limiting leadership groups’ ability to support one another effectively.

#### 3.4.2. Resources in the Work System Surrounding the Group and the Leader

In contrast, leaders viewed structural conditions as supportive when the work system helped manage demands and protect time for leadership. Several participants highlighted the importance of realistic spans of control and access to administrative support, which freed time for core leadership tasks. Control over scheduling and the ability to set clear boundaries were also emphasized as important resources. One leader described a simple yet significant practice: “Now I block my calendar 1 hour a day, with the door closed. It has helped me to get things planned.”

Others emphasized that respecting nonwork time is essential to sustained well‐being. One participant said, “My free time is also important to me – it must harmonize.” These practices were seen as more effective when supported by organizational norms and by immediate leaders who respected boundaries and modeled sustainable behavior. When these structural resources were available, leaders reported greater ability to prioritize, reflect, and focus on leadership activities, rather than being overwhelmed by administrative or operational pressures. The work system was therefore experienced not only as a background condition but also as part of how leaders described the functioning of leadership groups under pressure.

Across the four levels, participants described both risks and resources affecting nurse leader well‐being. The most recurrent risks were a lack of visible support from immediate leaders, low peer trust, limited access to confidential sparring outside the immediate group, high workload, role ambiguity, and expectations of constant availability. The most recurrent resources were visible managerial backing, clear priorities, psychological safety within peer leadership groups, coleadership or buddy arrangements, mentoring or cross‐unit sparring, administrative support, protected time, and manageable spans of control.

## 4. Discussion

The findings enhance understanding of nurse leaders’ well‐being by foregrounding how it is associated with relational and organizational conditions rather than by individual coping capacity alone. Across four relational and organizational levels—the immediate leader, the peer leadership group, the broader leadership network, and the surrounding work system—participants described risks and resources that affected their everyday leadership work. We interpret these findings as four interconnected resource patterns: vertical support and buffering, horizontal support and peer safety, cross‐boundary support, and organizational containment and manageable workload.

### 4.1. Theoretical Implications

Immediate leaders provided vertical support and buffering by clarifying priorities, involving nurse leaders in decisions, recognizing their professional judgment, and standing with them in critical situations. This aligns with research linking supervisory support, autonomy, influence, and organizational support to nurse leader well‐being, job satisfaction, and retention [2, 9, 22, 23]. Our findings suggest that this support was experienced as timely, visible backing that reduces leaders’ sense of exposure or of being weakly positioned in the hierarchy. At the same time, the findings show that involvement in decision‐making must be transparent. When priorities and opportunities were uneven or unclear, participants associated inclusion with tension among peers, undermining the resource it was meant to provide.

According to findings, peer leadership groups emerged as central yet ambivalent. When characterized by trust, confidentiality, daily sparring, and structured reflection, they contributed to horizontal peer safety and reduced isolation. When confidentiality was breached, conflicts became personal, or peers were too overloaded to offer support, the same group setting was described as intensifying loneliness and emotional strain. This ambivalence adds nuance to previous research identifying peer support, social support, and team cohesion as important resources for nurse leaders’ job satisfaction and well‐being [2, 14, 22]. It also suggests that peer support should not be treated as a generic resource and that its value is contingent on concrete group norms, sufficient time, and psychological safety.

Beyond the immediate peer group, broader leadership networks provided cross‐boundary reflection when leaders had access to mentors, HR/OD partners, external coaches, or cross‐unit peers. These resources were useful because they allowed leaders to test interpretations, normalize dilemmas, and discuss difficult issues with someone perceived as less involved in local relations. Some leaders, however, preferred individual sparring or highly trusted relationships over open group forums. This suggests that cross‐boundary support must offer varying degrees of confidentiality and exposure. The finding supports earlier work on the value of professional networks and organizational support and adds that distance from the local hierarchy may itself be an important resource [14, 15].

The surrounding work system provided structural containment when spans of control, administrative support, role clarity, protected time, and availability norms were connected to more manageable leadership demands. Conversely, the findings linked high workloads, administrative pressure, unclear expectations, and norms of constant responsiveness to less capacity among leaders to recover, reflect, and use relational support effectively. This aligns with studies identifying workload, role conflict, job strain, and span of control as key risks for nurse leaders’ stress and burnout, which may increase turnover intentions [4, 11, 21, 32]. The findings further indicate that organizational conditions do not merely coexist with relational resources but shape whether those resources can function. Even a supportive peer group may become insufficient when chronic overload leaves leaders without time or energy to engage.

These patterns extend the JD–R and COR perspectives by showing that nurse leader well‐being draws on resources distributed relationally and organizationally, not only on those held individually. In JD–R terms, the findings specify job resources at vertical, horizontal, cross‐boundary, and systemic levels. In COR terms, they show how resources may be created, shared, depleted, or replenished through everyday leadership relations and work‐system conditions [6, 17, 18]. This is important because interventions for nurse leaders’ well‐being have often emphasized individual stress management, whereas the present findings indicate that sustainable well‐being also requires collective and organizational resource‐building [13].

The findings also foreground the emotional burden of nurse leadership. Accounts of unresolved conflicts, difficult staff situations, and constant availability indicate that nurse leaders’ well‐being is challenged not only by workload and role strain but also by emotional and relational demands. This aligns with research on compassion fatigue, nurse leader stress, and the emotional intensity of healthcare leadership [11, 16]. Our findings suggest that these emotional demands become more manageable when leaders have access to trusted peers, confidential sparring, visible supervisory backing, and legitimate recovery boundaries.

Consequently, we conceptualize nurse leader well‐being as a relational and organizational capability: the shared capacity of leadership systems to buffer demands, replenish resources, and sustain leaders’ ability to lead under pressure. This capability is enacted through vertical support and buffering, horizontal support and peer safety, cross‐boundary support, and organizational containment. The contribution is not simply that support matters, but that it is organized and experienced across levels. Nurse leaders’ well‐being is therefore closely linked to the leadership infrastructure around the individual leader: supervisory practices, peer group norms, access to cross‐boundary reflection, and work‐system conditions that shape how demands are carried collectively rather than individually. This extends multilevel perspectives on psychosocial work environments by foregrounding the leadership group and its surrounding work system as key sites where demands and resources circulate [33]. It also responds to calls for leader well‐being research to move beyond the assumption that leaders should remain self‐reliant and resilient regardless of context [34].

### 4.2. Implications for Nursing Management

The findings point to several organizational focus areas for developing strategies to support nurse leaders’ well‐being. We translate the four resource patterns identified in the study—vertical support and buffering, horizontal support and peer safety, cross‐boundary support, and organizational containment and manageable workload—into implications for nursing management.

First, organizations should require and enable immediate leaders to set clear priorities, provide timely backing in critical situations, and ensure transparent involvement in decision‐making. This implication is grounded in participants’ accounts of feeling vulnerable when support was absent and more secure when their immediate leader was visible and accessible.

Second, organizations should support leadership groups as settings for confidential peer reflection, with explicit confidentiality norms. In this way, leadership groups could serve as forums for systematically addressing leadership dilemmas, workload pressures, and difficult relational situations. This may reduce isolation when leadership groups are grounded in trust and clear confidentiality norms. This implication is grounded in participants’ accounts of both peer safety and breaches of trust.

Third, organizations should ensure nurse leaders’ access to cross‐boundary sparring, such as mentoring, buddy arrangements, HR/OD support, external coaching, or cross‐unit communities of practice. This implication is grounded in participants’ descriptions of the need for perspectives beyond their own unit or immediate leadership group. Some participants found group openness challenging, so organizations should offer both group‐based and one‐to‐one formats.

Fourth, organizations’ focus on relational support should be paired with attention to structural conditions. Participants repeatedly described workload, administrative demands, role ambiguity, span of control, and expectations of constant availability as risks. Organizations should therefore regularly review and, where needed, adjust these conditions to reduce risks for nurse leaders’ well‐being and so that peer and supervisory support can function effectively in practice.

Based on the present study and prior research, we believe that these strategies will help sustain nurse leaders’ well‐being.

### 4.3. Future Research

Future research should examine whether the relational and structural focus areas identified in this study are associated with changes in nurse leaders’ stress, burnout, job satisfaction, and retention. Intervention studies could test peer reflection forums, mentoring or buddy arrangements, coleadership models, administrative support, and workload adjustments, using validated outcome measures and appropriate comparison groups. Longitudinal and multisite studies would be valuable for assessing whether these strategies have sustained effects on leaders’ well‐being across different healthcare settings.

Additionally, future research should include diverse settings and longer periods. Multisite or cross‐cultural studies can assess whether the same stressors and proposed interventions apply across different environments. Longitudinal designs are necessary to determine whether improvements in leader well‐being persist beyond the initial months. Extended follow‐ups (e.g., 12–24 months) would help confirm the durability of any benefits and whether ongoing support is needed. Research should also examine downstream effects, such as whether improving nurse leaders’ well‐being leads to better staff retention or patient outcomes, to strengthen the case for organizational investment. Expanding the scope and duration of studies will build a stronger evidence base to guide best practices.

### 4.4. Limitations

Although alignment with prior research suggests broad relevance, a context‐specific sample may not represent nurse leaders in other settings. The qualitative approach yielded rich, detailed perspectives. Although we thoroughly cross‐validated our findings across multiple interviews, the data were self‐reported, which may introduce bias.

The sample was drawn from one Danish healthcare region and comprised 18 nurse leaders and four other health managers who worked closely with nurse leaders in interprofessional leadership groups. This design supported analysis of relational leadership contexts but may limit transferability to settings with different professional compositions or governance structures. Despite these limitations, the study offers valuable exploratory insights.

We strengthened credibility through joint coding, cross‐interview comparisons, reflexive discussion, and participant quotations that illustrate each theme. Many of our findings align with broader research, reinforcing their credibility. However, additional research is needed to confirm these results across different settings and to evaluate the effectiveness of the proposed strategies. The limitations outlined here highlight the need for ongoing investigation of nurse leaders’ well‐being, using diverse methods and larger samples to build on the foundation established by this study. To strengthen the evidence base, future research should empirically test group‐level interventions and examine long‐term effects on nurse leader well‐being and staff outcomes.

## 5. Conclusion

The findings suggest that nurse leader well‐being is closely associated with specific relational leadership contexts rather than residing primarily within individual leaders. Across four interlinked levels (the immediate leader, peer leadership group, broader leadership network, and surrounding work system), participants described vertical support and buffering, horizontal peer safety, cross‐boundary support, and organizational containment as shaping whether high demands lead to sustainable leadership or to depletion. When these mechanisms were in place, leaders reported reduced isolation, greater control, and renewed capacity to lead. When they were absent, they reported feeling overloaded and vulnerable.

The findings, therefore, shift the intervention lens from individual coping to organizational and relational strategies that systematically enhance and protect nurse leaders’ well‐being. Accordingly, we conceptualize nurse leader well‐being as an organizational capability: a shared capacity to buffer demands through supportive supervisory relations, psychologically safe peer groups, and cross‐unit sparring structures, enabled by work systems that limit chronic overload and normalize sustainable boundaries. For executives and policymakers, this implies treating leader well‐being as integral to daily leadership work and to quality and safety governance, rather than as an optional add‐on.

## Author Contributions

Jan Heiberg Johansen and Malene Friis Andersen contributed to conceptualization, methodology, investigation, formal analysis, and interpretation. Jan Heiberg Johansen prepared the original draft. Both authors reviewed and edited the manuscript and accept responsibility for the work.

## Funding

This research received funding from the Danish Working Environment Authority.

## Disclosure

All authors approved the final version of the manuscript.

## Conflicts of Interest

The authors declare no conflicts of interest.

## Supporting Information

Additional supporting information can be found online in the Supporting Information section.

## Supporting information


**Supporting Information** The supplementary material consists of a COREQ checklist that summarizes how each of the 32 COREQ items is addressed in the study, providing an overview of the research team, study design, data collection, analysis, and reporting practices [26].

## Data Availability

The interview transcripts are not publicly available because the contextual detail in qualitative interview data could compromise participant confidentiality. Requests for access may be directed to the corresponding author and will be considered subject to participant consent, applicable data‐protection requirements, and institutional approval.
