# Supplementary material for: From Individual to Group: Organizational and Relational Strategies for Nurse Leader Well‐Being
Source: J Nurs Manag. 2026 Aug 23;2026:7568215. doi: 10.1155/jonm/7568215 (PMC13501005; doi:10.1155/jonm/7568215)
Supplement: Supplementary file 1 [file JONM-2026-7568215-s001.docx]

# Supplementary material. COREQ checklist

This checklist summarizes how each of the 32 COREQ items is addressed in the study, providing an overview of the research team, study design, data collection, analysis, and reporting practices (Tong et al., 2007).

| **No.** | **COREQ item** | **Information reported** |
| --- | --- | --- |
| **Research team and reflexivity** | | |
| 1 | Interviewer/facilitator | Interviews were conducted by the two authors. |
| 2 | Credentials | Both authors are trained, experienced researchers. |
| 3 | Occupation | The authors are researchers in organizational psychology, leadership, and work environment research. |
| 4 | Gender | Male and female. |
| 5 | Experience and training | Both authors have extensive experience conducting qualitative interview studies in healthcare and leadership contexts. |
| 6 | Relationship established | No prior personal or managerial relationship existed between the interviewer and the participants before the commencement of the study. |
| 7 | Participant knowledge of the interviewer | Participants were informed of the study's purpose, the researchers’ academic roles, and that participation was voluntary and confidential. |
| 8 | Interviewer characteristics (bias, assumptions) | The researchers’ background informed their attention to relational dynamics; potential interpretive bias was addressed through collaborative coding and reflexive discussions. |
| **Study design** | | |
| 9 | Methodological orientation and theory | The study used a qualitative, exploratory interview design informed by Job Demands-Resources (JD-R) and Conservation of Resources (COR) frameworks. |
| 10 | Sampling | Purposive sampling of 18 nurse leaders and four other health managers who worked closely with nurse leaders in interprofessional leadership groups within a Danish healthcare region. |
| 11 | Method of approach | Participants were invited via the regional nursing leadership network and volunteered. |
| 12 | Sample size | Twenty-two leaders participated, including 18 nurse leaders. |
| 13 | Non-participation | No enrolled participants withdrew. |
| 14 | Setting of data collection | One-to-one interviews were conducted virtually within the organizational context of a Danish regional healthcare system (primarily hospital and social service settings). |
| 15 | Presence of non-participants | No non-participants were present during interviews. |
| 16 | Description of sample | The sample comprised 18 nurse leaders and four other health managers who worked closely with nurse leaders in interprofessional leadership groups. Participants included first-line and middle-level nurse leaders from medical, surgical, psychiatric, and other departments; leadership tenure ranged from ~2 to 25 years (most 5-10 years); 14 women and 8 men. |
| 17 | Interview guide | A semi-structured interview guide was used, informed by prior research on nurse leader well-being and by the JD-R risk-resource distinction. |
| 18 | Repeat interviews | No repeat interviews were conducted. |
| 19 | Audio/visual recording | Interviews were audio-recorded. |
| 20 | Field notes | Notes were taken during interviews using a structured visual model capturing risks and resources in real time. |
| 21 | Duration | Interviews lasted approximately 60 minutes. |
| 22 | Data saturation | Saturation was assessed through ongoing interview summaries and author discussions of emerging codes. After approximately two-thirds of the interviews, no new major risk/resource categories or relational levels were identified. Subsequent interviews mainly refined and confirmed the themes. |
| 23 | Transcripts returned | Transcripts were not returned to participants for comment or correction. |
| **Analysis and findings** | | |
| 24 | Number of data coders | Two researchers independently coded a subset of transcripts; the full dataset was coded using an agreed coding scheme. |
| 25 | Description of the coding tree | Codes were grouped into themes under “Risk Factors” and “Resource Factors,” structured around relational and group levels. |
| 26 | Derivation of themes | Themes were derived inductively from the data, guided by the risk-resource framework. |
| 27 | Software | Office 365 was used to manage the data. |
| 28 | Participant checking | Participants did not provide formal feedback on findings. |
| 29 | Quotations presented | Verbatim participant quotations are presented to illustrate each theme; quotes were anonymized. |
| 30 | Data and findings consistent | Findings are grounded in interview data and consistently illustrated with participant quotations. |
| 31 | Clarity of major themes | Major themes are presented across four relational and organizational levels influencing nurse leader well-being. |
| 32 | Clarity of minor themes | Variations and less common experiences are described. |
